# Pollutant profile complexity governs wastewater removal of recalcitrant pharmaceuticals

**DOI:** 10.1093/ismejo/wrae033

**Published:** 2024-02-29

**Authors:** Marcel Suleiman, Natalie Le Lay, Francesca Demaria, Boris A Kolvenbach, Mariana S Cretoiu, Owen L Petchey, Alexandre Jousset, Philippe F-X Corvini

**Affiliations:** Institute for Ecopreneurship, FHNW University of Applied Sciences and Arts Northwestern Switzerland, 4132 Muttenz, Switzerland; Institute for Ecopreneurship, FHNW University of Applied Sciences and Arts Northwestern Switzerland, 4132 Muttenz, Switzerland; Institute for Ecopreneurship, FHNW University of Applied Sciences and Arts Northwestern Switzerland, 4132 Muttenz, Switzerland; Institute for Ecopreneurship, FHNW University of Applied Sciences and Arts Northwestern Switzerland, 4132 Muttenz, Switzerland; Blossom Microbial Technologies B.V., Utrecht Science Park, Padualaan 8, 3584 Utrecht, The Netherlands; Department of Evolutionary Biology and Environmental studies, University of Zurich, 8057 Zurich, Switzerland; Blossom Microbial Technologies B.V., Utrecht Science Park, Padualaan 8, 3584 Utrecht, The Netherlands; College of Resources and Environmental Science, Key Lab of Organic-Based Fertilizers of China and Jiangsu Provincial Key Lab for Solid Organic Waste Utilization, Nanjing Agricultural University, 210095 Nanjing, China; Institute for Ecopreneurship, FHNW University of Applied Sciences and Arts Northwestern Switzerland, 4132 Muttenz, Switzerland

**Keywords:** bioremediation, bacterial pollutant-removal, wastewater, pharmaceuticals

## Abstract

Organic pollutants are an increasing threat for wildlife and humans. Managing their removal is however complicated by the difficulties in predicting degradation rates. In this work, we demonstrate that the complexity of the pollutant profile, the set of co-existing contaminants, is a major driver of biodegradation in wastewater. We built representative assemblages out of one to five common pharmaceuticals (caffeine, atenolol, paracetamol, ibuprofen, and enalapril) selected along a gradient of biodegradability. We followed their individual removal by wastewater microbial communities. The presence of multichemical background pollution was essential for the removal of recalcitrant molecules such as ibuprofen. High-order interactions between multiple pollutants drove removal efficiency. We explain these interactions by shifts in the microbiome, with degradable molecules such as paracetamol enriching species and pathways involved in the removal of several organic pollutants. We conclude that pollutants should be treated as part of a complex system, with emerging pollutants potentially showing cascading effects and offering leverage to promote bioremediation.

## Introduction

The widespread use of pharmaceuticals in society and agriculture as well as their unintentional release from production sites has led to an alarming increase in their presence and accumulation in wastewater treatment plants [1-3]. Organic pollutants pose a significant environmental concern due to their multiple and still poorly understood impacts on ecosystems and human health [4, 5]. Consequently, there is a growing need to understand and mitigate the impact of these diverse pollutants on biological processes in wastewater. Traditionally, research efforts have focused on studying the removal efficiency of individual/single pharmaceutical pollutants by microbial communities, which led to a classification of easily biodegradable, such as paracetamol [6-8], and recalcitrant micropollutants, such as ibuprofen and diclofenac [8-12]. Although these studies provided valuable insights into the degradation potential of specific compounds, they overlook the complexities of real-world scenarios, where several micropollutants co-occur [13, 14]. Here, we assess whether the complexity of the pollutant profile, the set of anthropogenic molecules present in a given environment, can determine the removal of the pollutants present.

A growing number of studies have highlighted the unpredictable effects of multiple environmental pressures on ecosystems` functioning, including microbes [15, 16]. Such unpredictable effects are also likely to happen within a pollutant profile: when microbial catabolic pathways overlap for specific pollutants, the enrichment of organisms degrading a compound may also promote the degradation of other harboring similar chemical patterns [17-19]. In addition, induction of “promiscuous” enzymes with a large substrate spectrum may lead to broader pollutant removal [20, 21]. Further important mechanisms within a pollutant profile can probably be cross-feedings [22, 23], in which certain microorganisms form degradation metabolites to sustain other microorganisms’ growth, co-metabolism [24, 25], and the transformation of a non-growth substrate in the obligate presence of a growth compound [26].

To gain first insights into the impact of the presence of several pharmaceuticals on their removal in wastewater, we exposed wastewater samples to a combinatorial mixture of one to five commonly detected pharmaceuticals. We measured pollutant removal and bacterial community compositions. We hypothesize that due to pollutant–pollutant and pollutant–microbe interaction within the pollutant profile, recalcitrant pollutants can be more efficiently degraded.

## Materials and methods

### Preparation of synthetic wastewater batch cultures

We set up a total of 96 batch cultures with a volume of 20 ml each (100-ml Erlenmeyer flasks). Each culture contained as a basis synthetic wastewater, following the OECD standard procedures (0.08-g/l peptone, 0.05-g/l meat extract, 15-mg/l urea, 3.5-mg/l NaCl, 2-mg/l CaCl_2_ x 2 H_2_O, 0.1-mg/l MgSO_4_ x 7 H_2_O, and 1.4-mg/l K_2_HPO_4_, pH 7.5) (https://www.oecd.org/chemicalsafety/testing/43735667.pdf). Sterile filtered stock solutions of caffeine (C), atenolol (A), enalapril (E), paracetamol (P), and ibuprofen (I) were set up with a concentration of 10 g/l, and 200 μl were added to each batch culture, respectively, resulting in a final concentration of 100 mg/l of each pollutant. The five pollutants were added to the batch cultures in all possible combinations, leading to a total of 32 treatments (Table 1) (Supplementary Fig. 1 for chemical structures of the five pollutants). Each treatment was setup in triplicates.

**Table 1:** Summary of the combinations of pharmaceuticals in the batch cultures.

Compounds	Combinations	Combination possibilities
0	0	1
1	I, P, C, A, E	5
2	IP, IC, IA, IE, PC, PA, PE, CA, CE, AE	10
3	IPC, IPA, IPE, ICA, ICE, IAE, PCA, PCE, PAE, CAE	10
4	IPCA, IPCE, IPAE, ICAE, PECA	5
5	IPCAE	1

We used an additive design, meaning that each pollutant was added at a concentration of 100 mg/l. In addition, an abiotic control was set up in triplicate for each pollutant to ensure that no degradation occurred in absence of microorganisms. To prepare samples for inoculation, 50 ml of wastewater were taken from a wastewater treatment plant (Membrane bioreactor). The sample was shaken with 200 rpm for 15 minutes to achieve optimal homogenization. The batch cultures were inoculated with 200-μl sample (1% (v/v)), respectively. Before and after each inoculation, the wastewater treatment sample was homogenized again for 30 seconds. Incubation took place at 22°C for 11 days, under continuous shaking of the cultures (130 rpm).

To check whether the results remain valid over a range of pollutant concentrations, we included an additional experiment with batch cultures treated with E, PE, and IPCAE at individual concentration of 1 mg/l (100× less than in main experiment).

### Sampling

On Days 0, 3, 4, 7, and 11, one 500-μl sample was taken from each flask, centrifuged at 16 000× g for 5 minutes and the filtered (0.45-μm pore filter) supernatant was used for high-performance liquid chromatography (HPLC) analysis. In addition, on Days 3 and day 11, 2 ml of culture was taken and centrifuged at 16 000× g for 5 minutes, and the resulting pellet was taken for DNA extraction.

### HPLC analysis for measurements of pollutants

Pharmaceuticals were analyzed using HPLC with a ZORBAX RR StableBond C18 column (Agilent Technologies). The separation was achieved by applying a flow rate of 0.7 ml/min, using a mobile phase consisting of a mixture of water and methanol. Detection of the pharmaceuticals was performed using a UV/VIS DAD detector. The initial mobile phase ratio was set at 80:20 VV, comprising 0.1% formic acid in Millipore water (A) and methanol (B). The B gradient was programmed to transition from 20% to 95% over a span of 15 minutes, enabling simultaneous analysis of all five micropollutants in a single run. The retention times for each pharmaceutical were as follows: ibuprofen eluted at 14.23 minutes, enalapril at 11.08 minutes, caffeine at 7.97 minutes, atenolol at 2.11 minutes, and paracetamol at 2.89 minutes. Paracetamol, ibuprofen, atenolol, and caffeine were detected at a wavelength of 230 nm, whereas enalapril was detected at 205 nm. Standard curves were generated for each pollutant, ranging from 1 to 500 mg/l (1, 10, 50, 100, and 500 mg/l).

### DNA extraction and sequencing

We extracted DNA out of Days 3 and 11 samples for sequencing of the 16S rRNA gene. We selected these time points based on following rationales: On Day 3, the category 1 pollutants (paracetamol, atenolol, and caffein) were mostly degraded, so it was relevant to analyze the microbial communities at this point. At Day 11, pollutants of the category 2 (ibuprofen and enalapril) were mostly or partly degraded. DNA was isolated using the ZymoBIOMICS DNA Miniprep Kit (ZymoResearch, Irvine, USA) according to the manufacturer’s instructions. We sequenced the V4 region of the 16S rRNA gene (primer sequences 515f “GTGYCAGCMGCCGCGGTAA” and 806r “GGACTACNVGGGTWTCTAAT”) 27 using the Quick-16S Plus NGS Library Prep Kit (V4) (ZymoResearch) to create a DNA library. The library, containing 4 pM DNA (spiked with 25% PhiX), was sequenced in-house on a MiSeq platform (Illumina), following the manufacturer’s instructions. Raw reads were processed using the R library dada2 [27]. This involved quality control steps included analyzing primer sequences, assessing error rates (maxN = 0, maxEE = c(2,2), truncQ = 2), and identifying chimeras. The resulting sequence table (Min. number of reads = 187 722, Max. number of reads = 2 049 193, Total number of reads = 160 233 034, Average number of reads = 88 040) was aligned to the SILVA ribosomal RNA database [28] using version 138 (non-redundant dataset 99). A phyloseq object was then created using the phyloseq R library [29] . This object consisted of an amplicon sequence variant table, a taxonomy table, and sample data. Functional gene profiles of microbial communities were inferred using PICRUSt2 [30] and BioCyc [31] . Differential pathway enrichment was evaluated with deseq2 [32]. The phyloseq object, metadata, and detailed R code for analysis can be found on GitHub at https://github.com/Marcel2907. The raw sequencing data are available on the NCBI SRA (Sequence Read Archive) under the accession ID PRJNA1041291.

### Statistical analyses

A statistical model was used to analyze the degradation of each compound on each day. In each model, the response variable was the percentage remaining of the focal compound on a specific day. In each model, there were four binary explanatory variables, each of these coding the presence of the four non-focal compounds. All two-way and three-way interaction terms among explanatory variables were included, as well as the one four-way interaction. In all cases, the model was a linear model with Gaussian errors (model diagnostics were acceptable). Therefore, e.g., with paracetamol as the focal compound, the model in R would be

lm(P ~ I^*^C^*^A^*^E).

For heatmaps, the effect sizes for each day were calculated based on the summary of statistical analyses of pollutant interactions. Rows show the estimated coefficients of the single, one-way, two-way, three-way, and four-way interaction terms on pollutant concentration. White cells indicate a response variable and coefficient pairs for which the coefficients were not significantly different from zero (*t*-test *P*-value > .05), otherwise the diverging color palette illustrates the direction of the influence by the driver or interaction of drivers (based on the estimates of the *f*-test). A linear model was also used to analyze how microbial biomass, microbial diversity, and the relative abundance of three most abundant taxa depended on the five compounds and their interactions. All statistical analyses can be found in detail in Supplementary Tables 1 and 2.

## Results

### Removal kinetics of single and multiple pharmaceuticals in batch cultures

**Figure 1:**
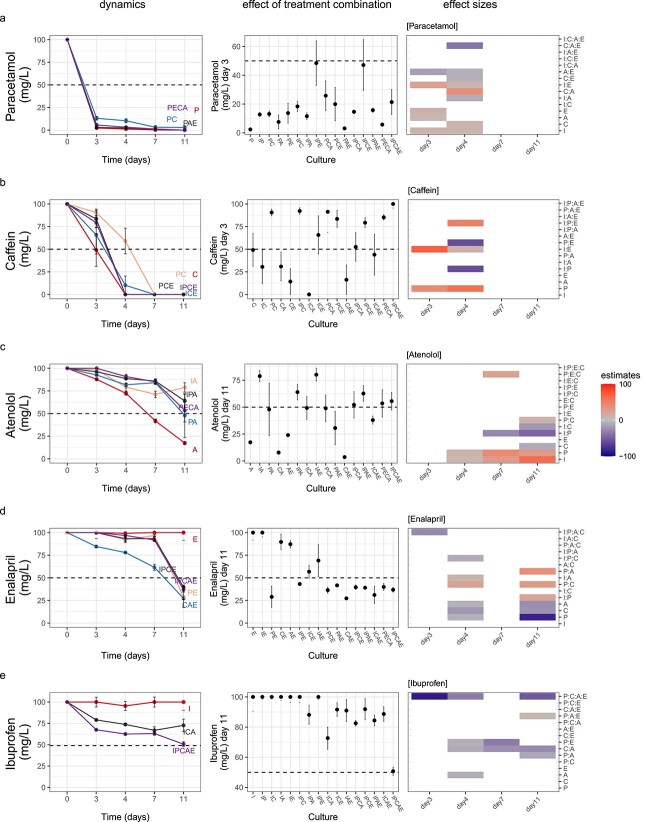
The presence of one or more pollutants increases the degradation of another pollutant, and each of the 5 pollutants appear in a row and the rows are sorted from least to most recalcitrant top to bottom; dynamics and effect sizes of pharmaceuticals concentration within the batch cultures dependent on the presence of additional pharmaceuticals; accentuation of the influence of pollutant combinations on the concentration of paracetamol (A), caffeine (B), atenolol (C), enalapril (D), and ibuprofen (E); error bars show ±SE (*n* = 3), and the effect sizes for each day are the summary of statistical analyses of pollutant concentrations (third column), and rows show the estimated coefficients of the single, one-way, two-way, three-way, and four-way interaction terms on pollutant concentration, and white cells indicate a response variable and coefficient pairs for which the coefficients were not significantly different from zero (*t*-test *P-*value >.05), otherwise the diverging color palette illustrates the direction of the influence by the driver or interaction of drivers, and positive estimates are meaning higher concentrations of pollutants (i.e. lower degradation), whereas negative estimates are meaning lower concentrations of pollutants (i.e. higher degradation); A: Atenolol, C:Caffein, E: Enalapril, I: Ibuprofen, P: Paracetamol.

Degradation rates of single pharmaceutical substances in isolation clustered them in two groups: Category 1 (degradable) encompassed caffeine, paracetamol, and atenolol. Caffeine and paracetamol were completely removed within 3–4 days, while about 80% of atenolol was removed within 11 days (Fig. 1A–C; Supplementary Fig. 2). The second category (recalcitrant) contained enalapril and ibuprofen, which were not degraded when individually present (Fig. 1D, E; Supplementary Fig. 2).

The degradation rates in mixed pharmaceuticals strongly departed from this baseline. The presence of degradable pharmaceuticals increased the degradation of ibuprofen and enalapril. For example, ibuprofen concentration was reduced to 50% of the original concentration when present alongside all four other pharmaceuticals, and to 70%–90% of the initial concentration in some combinations of two or three other compounds (Fig. 1E). Enalapril was reduced to ~30% of its initial concentration when paracetamol was also present and or when paracetamol was absent but both atenolol and caffein (“CAE”) were present (Fig. 1D).

Although pollutants of category 1 were to some extent degradable in all batch cultures, some inhibition effects were observed. For instance, atenolol degradation was hindered in the presence of ibuprofen or paracetamol, and by the presence of various other combinations of other compounds (Fig. 1C). In contrast, no striking additive negative effect was observed when atenolol was present alongside ibuprofen and paracetamol together (Fig. 1C “IPA”). Also, the removal of caffeine was slower in the presence of other pharmaceuticals, especially when paracetamol was present a long lag-phase occurred (Fig. 1B). In contrast, paracetamol degradation was only marginally inhibited by the presence of caffeine (Fig. 1A).

These effects of the presence of combinations of other pharmaceuticals on the degradation of a pharmaceutical were not only visually clear but were also clearly revealed by statistical analyses (Fig. 1 third column, Supplementary Table 1). These analyses revealed strong evidence of two-way, three-way, and four-way interaction effects and dependencies among pollutants in the removal processes for all tested pharmaceuticals and were statistically confirmed.

**Figure 2:**
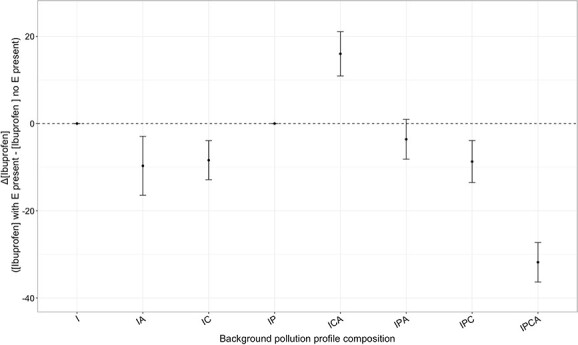
Pollutant profile complexity impacts the effect of additional pollutants on the biodegradation process; this figure shows the effect of enalapril addition on the biodegradation rate of the recalcitrant pollutant ibuprofen, and pollutant profile complexity is defined here as the number and identity of pharmaceuticals in the mixture; impact of enalapril is defined as the average difference in ibuprofen concentration in a mix of pollutant with or without enalapril; enalapril addition had little effect on the biodegradation of ibuprofen alone (I), but strongly increased its removal when all other pollutants were present (IPCA). A: Atenolol, C:Caffein, E: Enalapril, I: Ibuprofen, P: Paracetamol, and error bars show ±SE (*n* = 3).

The findings suggest that introducing one/more specific pollutant/s significantly affects the degradation of another compound, with the outcome being influenced by the presence of other pollutants. Particularly in the case of ibuprofen removal, identified as the most recalcitrant substrate in this study, notable variations were observed depending on the presence or absence of enalapril (Fig. 2). For instance, the introduction of enalapril on top of IPCA background pollution profile led to a 30% increase in the removal of ibuprofen compared with cultures containing only IPCA and lacking enalapril (Fig. 2). Removal patterns of Enalapril in selected batch cultures subjected to a far lower pollutant concentration of 1 mg/l showed similar interactive effects between category 1 and 2 pollutants (Supplementary Fig. 3), indicating that interactions are dose independent.

### Microbial community composition dependencies on pharmaceutical combinations

**Figure 3:**
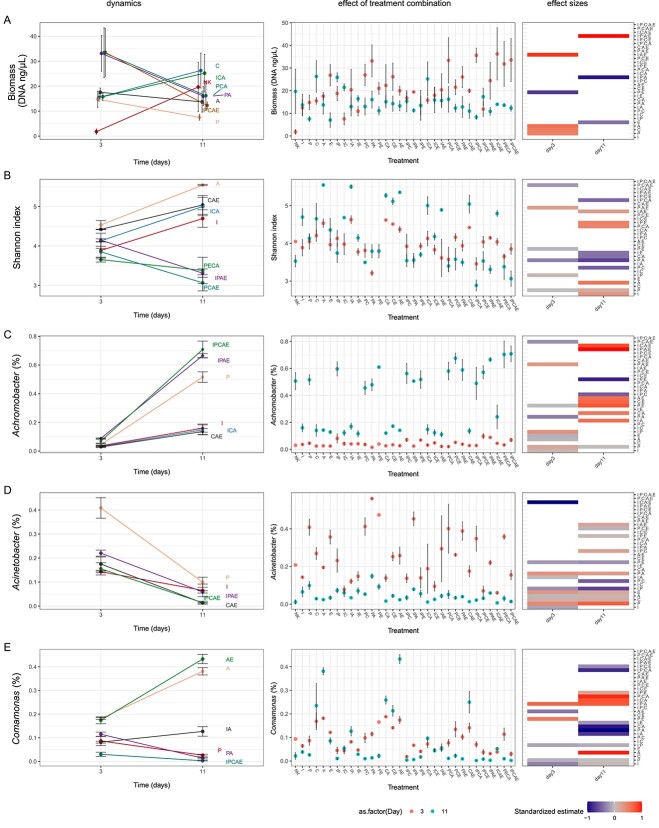
Dynamics and effect sizes of microbial community variables dependent on pharmaceutical combinations, and dynamics and effect sizes are showing the influence of pollutant combination on biomass (A), diversity (B), relative abundance of *Achromobacter* (C), *Acinetobacter* (D), and *Comamonas* (E); the effect sizes for each day are the summary of statistical analyses of pollutant concentrations (third column); rows show the estimated coefficients of the single, one-way, two-way, three-way, four-way, and five-way interaction terms on pollutant concentration; white cells indicate a response variable and coefficient pairs for which the coefficients were not significantly different from zero (*t*-test *P*-value > .05), otherwise the diverging color palette illustrates the direction of the influence by the driver or interaction of drivers (estimates of each variable were standardized by dividing by the largest absolute value of the estimates in each variable); NK = control (synthetic wastewater without addition of pharmaceuticals); A: Atenolol, C: Caffein, E: Enalapril, I: Ibuprofen, P: Paracetamol.

Biomass increased in all batch cultivations, indicating an active microbial community. On Day 3, biomass was significantly higher in the presence of single degradable pollutants (atenolol, caffeine, paracetamol) compared with pharmaceutical-free controls or single recalcitrant pollutants (ibuprofen, enalapril) (Fig. 3A, main effects in Supplementary Table 2). There was no clear relationship between the number of pollutants and microbial biomass (*f*-test *P-*value 0.57 (number of stressors as a factor)). There is perhaps a pattern of higher biomass when certain combinations of pollutants are present (i.e. PA, PCA, IPCA, ICAE, PECA, and IPCAE). Furthermore, while ibuprofen had uniquely high degradation in the IPCAE treatment, that treatment did not have uniquely high microbial biomass (e.g. the background community ICAE without or with P had similar microbial biomass, statistics in Supplementary Table 2), suggesting at least for ibuprofen that differences in microbial biomass did not drive the observed difference in degradation. Depending on the combination of pharmaceuticals, the biomass mostly decreased from Days 3 to 11, with some exceptions (Fig. 3A, “C,” “ICA”).

Shannon index, the reference index used to depict biodiversity, was strongly impacted by pharmaceutical treatment combinations (Fig. 3B). Furthermore, the presence of atenolol increased Shannon index in all cultures, regardless of the number of pharmaceuticals present (main effect of atenolol Supplementary Table 2).

**Figure 4:**
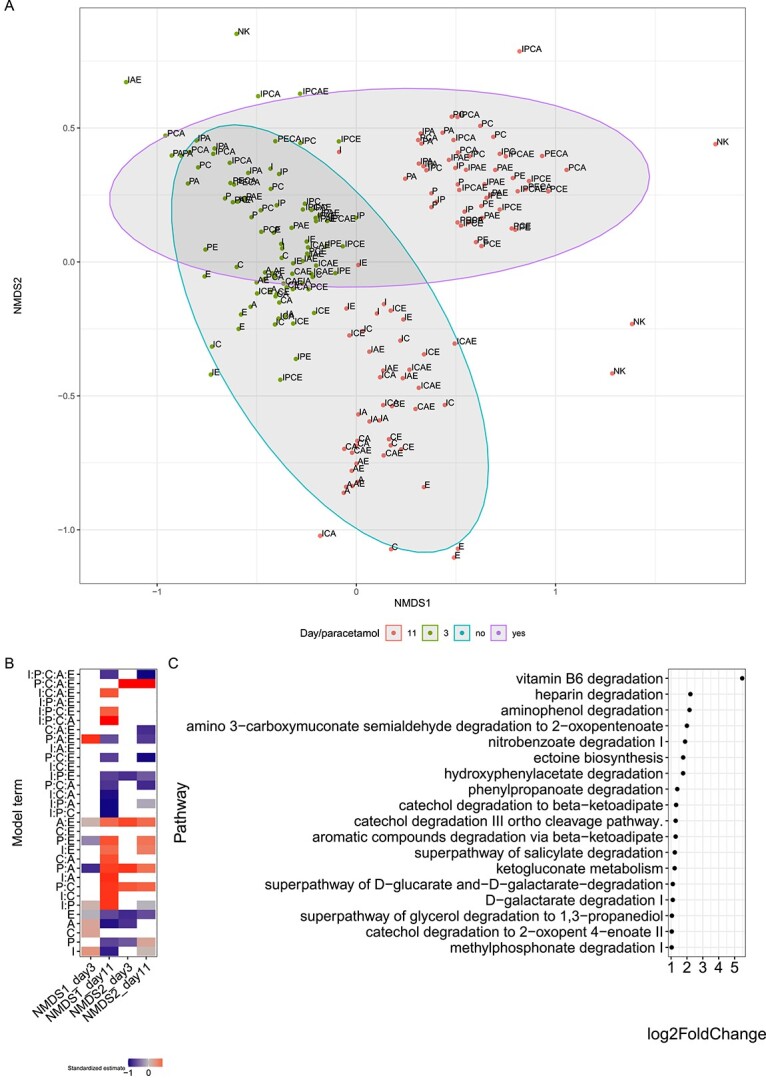
Effect of pharmaceutical combinations on microbial community structure and function, and (A) beta-diversity based on NMDS analysis (Bray–Curtis) in relation to the number of micropollutants present and day, and all samples containing paracetamol on Day 11 are clustering on the top right. Stress is 0.17; (B) the effect sizes for each day are the summary of statistical analyses of pollutant concentrations for NMDS1 and NMDS2; rows show the estimated coefficients of the single, one-way, two-way, three-way, and four-way interaction terms on pollutant concentration; white cells indicate a response variable and coefficient pairs for which the coefficients were not significantly different from zero (*t*-test *P-*value > .05), otherwise the diverging color palette illustrates the direction of the influence by the driver or interaction of drivers (estimates of each variable were standardized by dividing by the largest absolute value of the estimates in each variable); NK = control, no pollutant added (just synthetic wastewater); (C) overview of the MetaCyc pathways (*P*-value < .01) overrepresented in microbial communities grown on a pollutant profile containing paracetamol relative to those grown on pollutant profiles without paracetamol, and pathway enrichment was performed with deseq2 on the PICRUSt2 – Inferred functional gene profiles; the most significant affected pathways are shown; A: Atenolol, C: Caffein, E: Enalapril, I: Ibuprofen, P: Paracetamol.

The microbial communities showed strong differences depending on the pharmaceutical treatment on Days 3 and 11. On Day 3, genera with high relative abundance within the microbial communities were identified as members of the genera *Achromobacter*, *Trichococcus*, *Acinetobacter*, *Pseudomonas*, and *Comamonas*, whose abundance strongly varied in different treatments (Fig. 3C–E, Supplementary Fig. 5). For example, *Trichococcus* showed a relative abundance of 17–27% in triplicates incubated with PECA but was below detection when cultivated with enalapril (E) only. Comparable treatment effects were observed for these genera on Day 11, but in different strengths (Fig. 3C–E, second column). Additionally on Day 11, the proportion of members of the genus *Achromobacter* strongly increased. This was also highly influenced in their magnitude dependent on the treatment combination (Fig. 3C, Supplementary Fig. 4). *Achromobacter* abundance was consistently increased by the presence of paracetamol, an effect reflected by non-metric multidimensional scaling analysis (NMDS), which showed a strong clustering of paracetamol cultures on Day 11 (Fig. 4A). On Day 11, paracetamol was already degraded (within 3 days). Therefore, *Achromobacter* may possibly be associated with the consumption of degradation products of paracetamol such as aminophenol which occurrence was verified by HPLC. Statistical analysis confirmed significant changes of relative species abundance caused by different combinations of pharmaceuticals (Fig. 3C–E, third column). *Achromobacter* abundance also increased in the negative control (no pollutant added), but NMDS indicates a different community composition in these controls compared with paracetamol-containing cultures (Fig. 4A, B, Supplementary Fig. 4, Supplementary Table 2).

Since microbial communities incubated with paracetamol exhibited a different community than all other treatments (Day 11; Fig. 4A), we compared their inferred functional gene profiles (Fig. 4C) using PICRUSt2 [30]. Paracetamol significantly enriched pathways for aminophenol degradation, catechol degradation, as well as several aromatic degradation pathways (Fig. 4). As these pathways are likely involved in the degradation of a broad range of organic molecules, their increase may explain the positive effect of paracetamol on other more recalcitrant pharmaceuticals.

## Discussion

Efficient pollutant removal from wastewater is essential for environmental safety, yet current water treatment facilities fail to remove organic pollutants such as pharmaceuticals [33]. Steering microbial communities within these unique ecosystems may be key to designing better removal strategies. The composition and function of microbial communities can rapidly change depending on the incoming water composition [34]. Therefore, these wastewater treatment plants can be seen as a model system for studying multiple drivers on microbial communities and their degradation capacity of pollutants. Pollutant removal has been extensively studied in isolation, providing detailed insights into the molecular mechanisms underlying biodegradation. However, these findings only marginally translate to real-world scenario, where multiple drivers co-occur [35, 36].

In this work, we shed light on the interactive effects of pollutants within the pollutant profile, using mixtures of pharmaceutical varying in biodegradability as a model. We demonstrate that the complexity of the pollutant profile is a major driver of biodegradation and that the presence of multichemical background pollution was essential for the removal rates of recalcitrant molecules. We found in particular the degradation of recalcitrant pollutants to be strongly modulated by the presence of other pollutants. Easily degradable pharmaceuticals such as paracetamol, atenolol, and caffeine enable the degradation of the more recalcitrant ibuprofen and enalapril. In addition, some pollutants may hinder the biodegradation of other. This was particularly striking for atenolol, which degradation was inhibited in the presence of ibuprofen. These two chemicals show strong structural similarities (e.g. benzene ring and alkyl chain), which might inhibit enzymatic activity. Ibuprofen deserves special attention, as it proved to be only degradable when incubated alongside other pharmaceutical compounds. This observation underscores the significance of studying the environmental fate of pharmaceuticals as a collective group rather than at single compound level.

Interactions between pollutants are likely due to shifts in microbial community composition and function. We identified a range of potential key players (based on high relative abundances) involved in pharmaceutical removal, namely *Achromobacter*, *Pseudomonas*, *Acinetobacter*, *Comamonas*, and *Trichococcus*. All of them have already been shown to be associated with pollutant degradation [6, 7, 37-40]. These genera strongly respond to the composition of the pollutant mix, potentially explaining previous observations of their fluctuations in wastewater treatment systems [41, 42]. In particular, paracetamol had a strong effect on the microbial community composition. This may be ascribed to the release of aminophenol, a broad-spectrum antimicrobial molecule, during the breakdown of paracetamol [7, 43]. In line with this hypothesis, paracetamol-treated cultures showed an increased abundance of the paracetamol degradation pathway and turned to a brownish color typical for aminophenol. Despite of potential toxicity, paracetamol addition led to an increase degradation of other pharmaceuticals. The paracetamol degradation pathway encompasses a high enzymatic diversity [7] and may also be involved in the degradation of other recalcitrant pharmaceuticals. One caveat to mention here is the fact that due to the comparatively high concentration used in this study, it is possible that the microbial community was not able to degrade fast enough the formed aminophenol, which might have accumulated transiently.

The major question which needs to be tackled in future studies is the reason for this observation. Potential mechanisms may include cross-feeding, elevated enzyme activity, increased energy levels, and the induced expression of genes encoding promiscuous enzymes catalyzing the degradation of more than one class of pollutant [20-23, 26]. As a guideline for further studies, the presented results likely rule out increased biomass as a contributing factor given the fact that ibuprofen-degrading cultures did not yield more biomass than other treatments. Co-metabolic effect related to enzyme that fortuitously accept various chemically related substrate could play a role, since the chemical structures of the used pharmaceuticals show some chemical similarities, such as aromatic rings (Supplementary Fig. 1). Therefore, it could be possible that, e.g. specific dioxygenases that play a role in paracetamol degradation [7, 44], could potentially also show (low) activity against ibuprofen and enalapril.

All tested pharmaceuticals are globally detectable in wastewater influents and effluents and occur worldwide in ng/l to μg/l scale [6, 45-48], which is significantly lower compared with the high concentration we used in this study. However, the CODs used in this study can occur in wastewater of industrial production sites of pharmaceuticals [49]. In this work, we opted for an additive design to reflect that in a real-world situation, the concentration of individual pollutants does not directly depend on the presence of other pollutants. However, we would like to stress that this design may entangle the effects of pollutant concentration and the number of pollutants present in a sample. To rule out concentration effects, we re-ran the experiment with selected batch cultures and an individual pollutant concentration of 1 mg/l. We observed a similar interaction pattern between the recalcitrant Enalapril and the easily degraded Paracetamol.

This study indicates that the presence of easily degradable micropollutants, such as caffeine, atenolol, and paracetamol, promoted the degradation of recalcitrant substrates such as ibuprofen and enalapril. In contrast, these latter compounds were not degraded when present as the sole pollutant. The significance of these discoveries is noteworthy, as they can serve as potential starting points for the development of future applications aimed at the effective removal of pharmaceuticals: the study demonstrated that the addition of specific compounds at specific time points can enhance the degradation of a target pollutant. Addition of non-toxic functional mimics of existing pollutants may thus improve the microbial removal of persistent pollutants, contributing to safe water, ecosystems, and food supply. We conclude that pollutants should be treated as part of a complex system, with emerging pollutants potentially showing cascading effects and offering leverage to promote bioremediation.

## Supplementary Material

Supplements_wrae033

## Data Availability

All datasets and metadata are available on the GitHub repository Marcel 29071989 (https://github.com/Marcel29071989/), and the raw sequencing data can be found on NCBI SRA archive under ID PRJNA1041291.

## Code availability

R Studio code for the analysis and plotting of figures for the manuscript and supplementary information is available at https://github.com/Marcel29071989
