# Correction to: Pollutant profile complexity governs wastewater removal of recalcitrant pharmaceuticals

**DOI:** 10.1093/ismejo/wrae222

**Published:** 2024-12-04

This is a correction to: Marcel Suleiman, Natalie Le Lay, Francesca Demaria, Boris A Kolvenbach, Mariana S Cretoiu, Owen L Petchey, Alexandre Jousset, Philippe F-X Corvini, Pollutant profile complexity governs wastewater removal of recalcitrant pharmaceuticals, *The ISME Journal*, Volume 18, Issue 1, January 2024, wrae033, https://doi.org/10.1093/ismejo/wrae033

The originally published version of this manuscript contained an error in the European Union Project NYMPHE grant ID number. This has been corrected from 10106625 to 101060625.

